# The Homeostatic Theory of Obesity: An Empirical Verification of the Circle of Discontent with an Assessment of Its Relationship to Restrained and Uncontrolled Eating among Children and Adolescents

**DOI:** 10.3390/ijerph17176028

**Published:** 2020-08-19

**Authors:** Kamila Czepczor-Bernat, Anna Brytek-Matera, Paweł Matusik

**Affiliations:** 1 Faculty of Psychology in Katowice, SWPS University of Social Sciences and Humanities, 40-326 Katowice, Poland; 2 Institute of Psychology, University of Wroclaw, 50-527 Wroclaw, Poland; anna.brytek-matera@uwr.edu.pl; 3 School of Medicine in Katowice, Department of Pediatrics and Pediatric Endocrinology, Medical University of Silesia, 40-752 Katowice, Poland; pmatusik73@gmail.com

**Keywords:** childhood obesity, the homeostatic theory of obesity, the circle of discontent, children, adolescents, body dissatisfaction, negative affect, restrictive eating, uncontrolled eating, high-energy consumption

## Abstract

The purpose of the present study was to provide an empirical verification of the Circle of Discontent with an assessment of its relationship to restrained and uncontrolled eating among children and adolescents. This study examined whether our results confirm a new hypothesized model. The total sample comprised 282 children and adolescents (148 girls and 134 boys; 141 participants with normal body weight and 141 with obesity). The mean age was 12.23 years (*SD* = 2.80), and the average BMI (body mass index) was 23.29 kg/m^2^ (*SD* = 6.27). The following were used: Positive and Negative Affect Scale for Children, Children’s Body Image Scale, Figure Rating Scale, Three-Factor Eating Questionnaire and Eating Disorders in Youth. The obtained values of the model fit indices proved the goodness of fit. Our findings show that obesity accompanies body dissatisfaction and uncontrolled and restrictive eating. Moreover, the higher the level of restrictive eating, the lower the level of uncontrolled eating. The relationships between body dissatisfaction, negative affect and restrictive eating, as well as that between uncontrolled eating and high-energy consumption, are significant and positive. Other relationships are non-significant. The above-mentioned relationships established in the Circle of Discontent, as well as relationships of restrained and uncontrolled eating with variables described in the circle, were confirmed. Based on our results, preventive strategies and psychological interventions can be created and may include changes in body image, eating behaviors and emotional functioning.

## 1. Introduction

Obesity has become a global epidemic in developed, as well as developing countries [1,2]. In Poland, the prevalence of obesity is worryingly high [3,4]. A recent study showed that the prevalence of obesity (with 95% CI) in the 7–18 age group was 4.3–8.8% among boys and 2.7–4.2% among girls [3].

**Figure 1:**
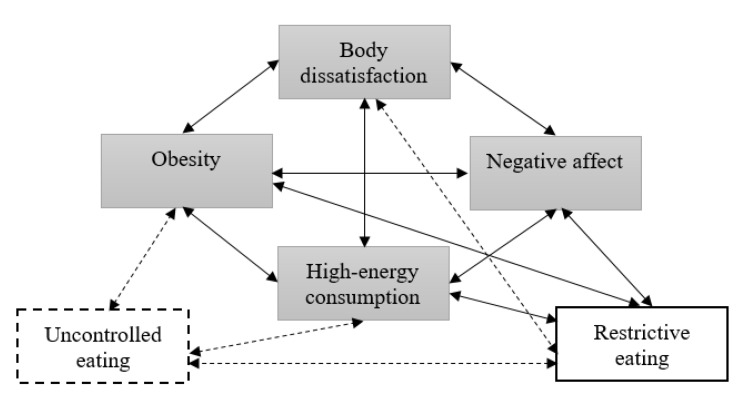
The Circle of Discontent (COD) and its relationship to restrained and uncontrolled eating among children and adolescents: a hypothesized model. 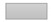 the variables from the Circle of Discontent; 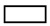 a variable from Homeostatic Theory of Obesity whose relationship with COD was described by Marks; 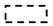 a new variable (proposed to be tested by the authors); 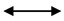 relationships described by Marks; 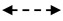 new relationships (proposed to be tested by the authors).

Obesity is considered a multi-factorial disease, and multi-component interventions are more effective than single-component interventions in reducing body weight [1,4]. It is therefore important to consider obesity from an interdisciplinary theory- and evidence-based perspective. One such approach is the Homeostatic Theory of Obesity [5]. This model was created by Marks [5] and is based on a homeostatic theory of physical and mental health and illness. Marks [5] assumes that obesity is at least in part an effect of homeostatic imbalance in psychological functioning. By providing considerable scientific evidence, Marks created the Homeostatic Theory of Obesity, in which the Circle of Discontent (COD) is considered particularly important. This vicious circle describes the relationship between obesity, high-energy consumption, negative affect and body dissatisfaction. One of the variables listed in the model, related to the Circle of Discontent, is restraint (identified by restrictive eating). All these interrelationships have been widely researched in many studies [5]. Based on these new outcomes [6,7,8,9,10,11,12,13], a new variable (uncontrolled eating) and new relationships (between uncontrolled and restrictive eating and other variables) were added to the model (Figure 1). These studies show that body mass index (BMI) [7,10] and high-energy consumption [6,9,12] increase as uncontrolled eating increases. Moreover, a high level of restrictive eating is accompanied by a low level of uncontrolled eating [13] and a high level of body dissatisfaction [8,10,11].

Based on Marks’ theory [5] and the other studies mentioned above, it was assumed that all relations (except for restrictive eating and uncontrolled eating as well as restrictive eating and high-energy consumption) are positive.

To the best of our knowledge, the model has not yet been empirically verified in research. Therefore, the purpose of the present study was to provide an empirical verification of the Circle of Discontent with an assessment of its relationship to restrained and uncontrolled eating among children and adolescents. This study examined whether our results confirm a new hypothesized model.

## 2. Materials and Methods

### 2.1. Participants

We focused on children and adolescents because obesity in these groups is a growing public health problem—the prevalence of obesity increased several times among both girls and boys between 1975 and 2016 [2]. Moreover, in obese children and adolescents, an increased risk of being obese in adulthood has been observed [1]. Seeking new information on obesity can help increase effectiveness in preventing and treating obesity at an early stage of development.

**Figure 2:**
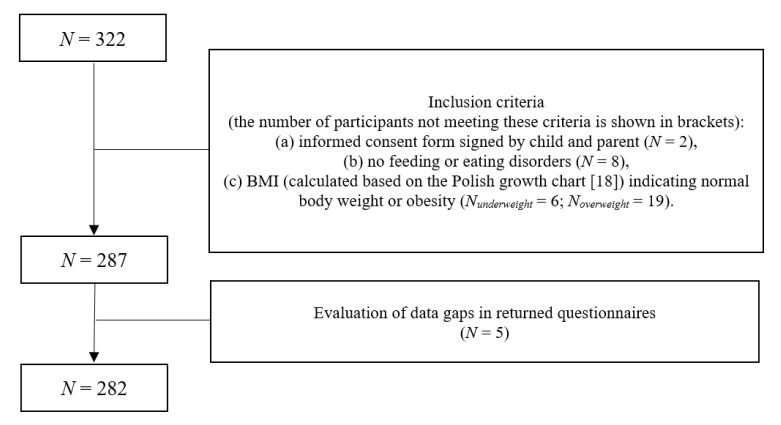
Flow chart of participants.

Information about the study was passed on to parents and their children by doctors, nutritionists and teachers. Three hundred twenty-two participants applied for the study (Figure 2).

The total sample comprised 282 children and adolescents (148 girls and 142 boys; 141 participants with normal body weight and 141 with obesity). The mean age was 12.23 years (*SD* = 2.80), and the average BMI was 23.29 kg/m^2^ (*SD* = 6.27). Based on the percentile analysis (BMI) [14], the participants were divided into two groups: normal body weight and obesity. Table 1 shows the characteristics of the participants.

### 2.2. Measures

Questionnaires that already had Polish validation and the reliability of which had been assessed at an earlier stage of the project (the first stage of longitudinal research, the National Science Centre, Poland, no. 2017/25/N/HS6/00004) were used.

#### 2.2.1. Negative Affect: Positive and Negative Affect Scale for Children (PANAS-C)

The Positive and Negative Affect Scale for Children (PANAS-C) is a self-report instrument measuring negative affect (NA) and positive affect (PA) among children and adolescents [16]. In the present study, a shorter 10-item Polish version was used, including (a) NA: “sad”, “scared”, “miserable”, “afraid”, and “mad”; (b) PA: “happy”, “cheerful”, “proud”, “joyful”, and “lively” [16]. The participants were asked, “How often have you felt this way during the past few weeks?” and responded on a 5-point scale (from 1—“not much or not at all” to 5—“a lot”) [16,17]. The higher the scores are, the higher the intensity of negative and positive affect. Cronbach’s alpha reliability (internal consistency) coefficients were (a) NA: 0.70 and (b) PA: 0.84.

#### 2.2.2. Body Dissatisfaction: Children’s Body Image Scale (Children) and Figure Rating Scale (Adolescents)

These scales are self-applied tools assessing the level of body dissatisfaction based on the discrepancy between one’s ideal and real self [18,19,20]. These questionnaires contain seven hand-drawn children’s and adolescents’ silhouettes (from the thinnest to the fattest) [18,19,20]. The participants were asked to answer two questions: (a) one’s real self: “Mark the red circle as you look now” and (b) one’s ideal self: “Mark the green circle to show how you would like to look”. We calculated body dissatisfaction by subtracting the number of the silhouette indicated as “one’s real self” from the number of the silhouette indicated as “one’s ideal self“.

#### 2.2.3. Uncontrolled and Restrictive Eating: Three-Factor Eating Questionnaire (TFEQ-R13)

This is a brief 13-item Polish version of a self-report measure [21]. This questionnaire assesses three types of maladaptive eating: (a) uncontrolled eating—“I am always hungry; therefore, I cannot stop eating until I empty my plate”; (b) restrictive eating—“I take small portions on purpose in order to control my body mass”; (c) emotional eating—“I eat when I feel nervous”. In the first twelve items, the participants responded on a 4-point scale (from 1—“definitely false” to 4—“definitely true”). The last item (13) used an 8-point scale (from 1—“eating whatever you want, whenever you want it” to 8—“constantly limiting food intake and never ‘giving in’”). This answer was recorded on a 4-point scale (1–2 into 1; 3–4 into 2; 5–6 into 3; 7–8 into 4). The higher the score, the higher the maladaptive eating style. In the current sample, Cronbach’s α (internal consistency) was (a) uncontrolled eating: 0.76; (b) restrictive eating: 0.87; (c) emotional eating: 0.88.

#### 2.2.4. Anthropometrical Status Based on BMI (Normal Body Weight vs. Obesity) and High-Energy Consumption: Sociodemographic Survey and Clinical Diagnosis

The sociodemographic survey included questions about sex, age, weight, height and high-energy consumption. In the case of younger children, parents helped with the measurement of weight and height. BMI was calculated based on self-report data. However, the diagnosis of obesity had to be confirmed by a doctor and/or dietitian. The participants were divided into two groups based on the percentile analysis (the calculation of children’s and adolescents’ body mass index using the Polish growth chart—the “OLAF” and “OLA” projects) [14]; thus, the variable included in the analyzed model was dichotomous—normal body weight (cut off points: 10–85th BMI percentile) vs. obesity (cut off point: above 95th BMI percentile). Additionally, to assess the level of high-energy consumption, the participants were asked “How often do you eat high-calorie snacks during the day (e.g., sweets, fast food, chips, salt sticks)?” (responses ranged from 1—“once a day or less often” to 6 “six or more times a day”).

#### 2.2.5. Feeding and Eating Disorders: Eating Disorders in Youth—Questionnaire (EDY-Q)

This brief self-report 14-item tool was used to exclude children and adolescents with symptoms of feeding and eating disorders [22]. This questionnaire was created based on the Diagnostic and Statistical Manual of Mental Disorders, Fifth Edition (DSM-5) [23].

### 2.3. Procedure

The participants were recruited in primary schools and centers specializing in the treatment of obesity (inpatient/outpatient) in the Opolskie and Silesian voivodeships between2018 and 2019. Before the participants completed the questionnaires, consent was obtained from them and their parents. No compensation was offered to the participants. The study was approved by the local ethics committee (no. 50/03/2017).

### 2.4. Data Analysis

The Statistical Package for Social Sciences version 25.0 with AMOS was used to verify the Circle of Discontent and its relationship to restrained and uncontrolled eating among children and adolescents. The proposed conceptual model was tested using structural equation modelling [24]. The generalized least squares (GLS) technique was used to estimate the parameters in our model. Moreover, bootstrapping was performed (number of bootstrap samples = 200).

The overall goodness of fit was assessed by the chi-square statistic (χ^2^), degrees of freedom (df), comparative fit index (CFI), root mean square error of approximation (RMSEA) and standardized root mean square residual (SRMR). A chi-square with a p-value greater than 0.05 and χ^2^/df less than 2 suggested goodness of fit, as did CFI greater than 0.95, RMSEA less than 0.06 (with pclose > 0.05) and SRMR less than 0.08 [24]. Moreover, the Bayesian information criterion (BIC) and Akaike information criterion (AIC) indicated that the hypothesized models were substantially nearer to saturated than the independent models.

## 3. Results

### 3.1. Descriptive Statistics and Correlations

Descriptive statistics and correlations (Pearson’s r) are shown in Table 2.

### 3.2. Empirical Verification of the Circle of Discontent and its Relationship to Restrained and Uncontrolled Eating Among Children and Adolescents

The goodness-of-fit statistics are presented in Table 3. We determined the goodness of fit between the hypothesized model (Figure 1) and our data and the obtained values of the model fit indices proved the goodness of fit [24].

**Figure 3:**
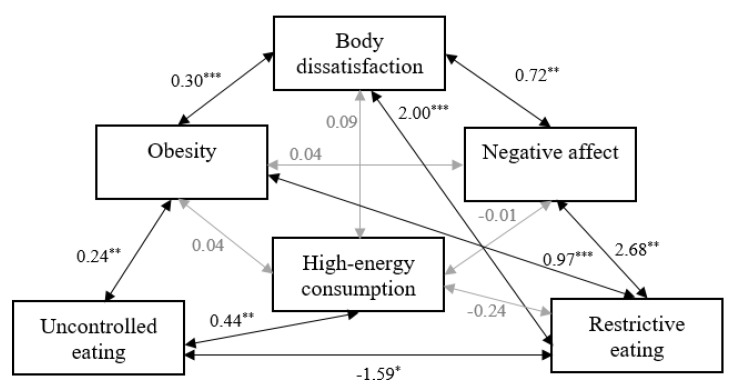
The Circle of Discontent and its relationship to restrained and uncontrolled eating among children and adolescents: a statistical model. * *p* < 0.05, ** *p* < 0.01, *** *p* < 0.001. The values for unstandardized coefficients are shown above the arrows. 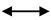 a significant relationship; 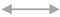 a non-significant relationship.

Based on Table 3, we conclude that the hypothetical relationships match the data well. Therefore, our results provide a basis for accepting the hypothetical model presented in Figure 1. Figure 3 captures the results (unstandardized estimates) with respect to the set of relations hypothesized by the researchers.

With regard to Figure 3, obesity accompanies body dissatisfaction and uncontrolled and restrictive eating. Moreover, the higher the level of restrictive eating, the lower the level of uncontrolled eating. The relationships between body dissatisfaction, negative affect and restrictive eating, as well as that between uncontrolled eating and high-energy consumption, are significant and positive. Other relationships are non-significant. Being obese is not related to the experience of negative affect and high-energy consumption. In addition, body dissatisfaction is not significantly associated with high-energy consumption, nor is high-energy consumption associated with negative affect and restrictive eating.

Additional information about the outcomes can be found on the website https://drive.google.com/open?id=10JfSwZRKss2ds5nk9nnaHa8n-DF3O0Ne.

## 4. Discussion

In the present study, we empirically verified the Circle of Discontent with an assessment of its relationship to restrained and uncontrolled eating among children and adolescents. To our knowledge, our evaluation is the first assessment of the relations assumed in the Homeostatic Theory of Obesity using structural equation modelling. Several key findings were revealed in the present work. Most of the relationships described in the hypothesized model are significant and consistent with the assumed direction.

Being obese co-occurs with experiencing body dissatisfaction and maladaptive eating behaviors in the form of uncontrolled and restrictive eating. Moreover, the association between restrictive eating and uncontrolled eating is significant and negative. The current findings are in line with previous studies demonstrating significant relationships between the above-mentioned variables [5,7,13]. A possible explanation for our outcomes is the current ideal of beauty. Numerous studies presented in Marks’ article [5] indicate that the current ideal of beauty is associated with having a thin silhouette [25]. Therefore, if there is a discrepancy between the current body weight and the ideal, there may be social pressure to change the appearance and strong body dissatisfaction. As a consequence, behaviors are undertaken to reduce body weight (e.g., restrictive eating) [5,25]. On the one hand, as everyone knows, these behaviors often do not have the desired effect because a restrictive diet often increases the risk of overeating (e.g., “masking hypothesis”) [5,26,27]. On the other hand, not all studies confirm the negative impact of restrictions on overeating [28,29] or show that this relationship is negative [13]. Therefore, further research into these relationships is necessary.

Consistent with previous findings [5,6,8,9,10,11], our findings indicate that the subsequent relationships between body dissatisfaction, negative affect and restrictive eating, as well as that between uncontrolled eating and high-energy consumption, are significant and positive. Therefore, as predicted by Marks [5], a high level of body dissatisfaction is associated with the experience of a high intensity of negative affect, and the source of these emotions can be difficulty in controlling eating behavior, a large discrepancy between current and ideal body weight, and discrimination on the basis of physical appearance and body weight. Negative affect can trigger emotional eating, which, if it becomes a constant way of regulating emotions, may lead to weight gain [5,30]. Therefore, people often use dietary restraint to prevent weight gain and reduce the level of body dissatisfaction, negative affect and uncontrolled eating [5,30]. Dietary restrictions are a particularly important method of weight control for people with a high level of uncontrolled eating because they often consume more total energy, snacks and desserts than controlled eaters [9,30,31]. This is because a tendency towards uncontrolled eating is associated with the consumption of high-calorie foods (e.g., sweets, chocolate, fast food), which can also lead to weight gain [32,33,34].

Other relationships have not been confirmed. Obesity is not associated with the experience of negative affect and high-energy consumption. In addition, the analysis shows that body dissatisfaction is not significantly related to high-energy consumption, nor is high-energy consumption associated with negative affect and restrictive eating. Interestingly, Marks [5] assumes that all these relationships are significant and positive (except for restrictive eating and high-energy consumption). However, we can find studies both confirming these assumptions [32,33,35,36,37], and contradicting them or showing that the relationship among the variables is weak [38,39]. Still, other studies indicate the need to include moderators and mediators (e.g., sex, stress, size of image on food packaging targeting children) of the relationships we analyze [40,41,42].

It should be noted here that almost all relationships between high-energy consumption and other variables are non-significant. Consideration should therefore be given to conducting a future study using a different questionnaire that will more validly and reliably assess high-energy consumption (more information is provided in the paragraph on limitations). Moreover, many of the studies presented in this article have been conducted in the adult population (and not among children and adolescents). Therefore, caution should be exercised in drawing conclusions. Future research can help clarify current inconsistencies in the literature.

Given the high costs of obesity and comorbidities in terms of healthcare expenditure, prevention strategies are much needed [43]. When planning actions, keep in mind that childhood obesity is a complex issue [4,44]. Interestingly, one of the reports of the World Health Organization on obesity among Polish children indicates the need to carry out activities directed at improving the mental functioning of obese children [4]. Therefore, for the effectiveness of such activities to be as high as possible, it is necessary to integrate knowledge from many disciplines (including medicine, dietetics, physiotherapy, psychology) [4].

### 4.1. Strengths

To eliminate the impact of a subjective assessment of obesity, children and adolescents with a diagnosis of obesity confirmed by a doctor and/or dietitian participated in the study. First empirical verification of the relations assumed in the Homeostatic Theory of Obesity, an evaluation of obesity as a complex issue and knowledge that can be helpful in a multidisciplinary approach to obesity are the strong points of this study.

### 4.2. Limitations

Our study has some limitations that should be taken into consideration in interpreting outcomes and planning further studies. First, due to the cross-sectional design of the study, causality cannot be inferred, and longitudinal studies are required. Second, the size of the sample in the present study (based on the standards for structural equation modelling; [24]) is relatively small. Third, our analysis assesses only a selected fragment of relations assumed in the Homeostatic Theory of Obesity (the Circle of Discontent) together with an assessment of the relationship to restrained and uncontrolled eating among children and adolescents. Fourth, the study does not include emotional eating, which, as it turned out, could be important for interpreting the relationship between negative affect and restrictive eating. Therefore, we plan (in the third stage of our research, no. 2017/25/N/HS6/00004) to evaluate all relationships between the variables described in the Homeostatic Theory of Obesity, taking into account both the new relationships presented in this study and a much larger number of participants as well as incorporating emotional eating into the model. Fifth, we used a measure of high-energy consumption based on a single item about the frequency of eating high-calorie snacks during the day. In the future, a valid and reliable questionnaire (e.g., Food Frequency Questionnaire) may be used. This will provide information on the frequency and/or portion size of different types of food and beverages (typically over the past month or year). Sixth and last, because in our study BMI was calculated based on self-reported data, it would be worth applying an objective assessment of weight and height to all participants using bioelectrical impedance analysis to estimate body composition.

## 5. Conclusions

Most of the relationships established in the Circle of Discontent, as well as relationships of restrained and uncontrolled eating with variables described in the circle, are confirmed among children and adolescents. The following relationships are significant and consistent with our assumptions: (a) positive relationships: (1) obesity and body dissatisfaction, (2) obesity and uncontrolled eating, (3) obesity and restrictive eating, (4) body dissatisfaction and negative affect, (5) body dissatisfaction and restrictive eating, (6) negative affect and restrictive eating, (7) uncontrolled eating and high-energy consumption; and (b) negative relationship: (1) uncontrolled eating and restrictive eating. As suggested in the Discussion and Limitations sections, the main findings of our research and all limitations should be included in the next stages of the study.

Based on our research, we might create prevention strategies and psychological interventions that involve: (a) decreasing body dissatisfaction; (b) reducing high-energy consumption and maladaptive eating styles (uncontrolled and/or restrictive eating); (c) decreasing negative emotions and increasing adaptive emotional regulation. This information can be combined with knowledge already available in the fields of medicine, dietetics and related disciplines. In the light of these and other findings, there can be little doubt that the addition of psychological components to interventions would lead to improvements in the functioning of children and adolescents with obesity. These proposed interventions can help to slow the obesity epidemic.

The mechanism of obesity development is still being explored [1]. A biopsychosocial approach is based on the belief that the causes of obesity are attributable to genetic, nutritional and environmental factors [4,45,46,47]. Our and previous research shows that an increased body mass index can also be associated with psychological factors (e.g., increased body dissatisfaction, restrictive eating and uncontrolled eating) [6,8,9,10,11]. The above-mentioned factors often form a vicious circle that hinders effective behavioral control and weight loss [4]. It is therefore necessary to create prevention strategies and psychological interventions that take this information into account.

## Figures and Tables

**Table 1:** Characteristics for the two BMI categories.

	Normal Body Weight *N* = 141	Obesity *N* = 141	
	*N* (%)		*p*
Sex 1			>0.05
Female	76 (53.90)	72 (51.06)	
Male	65 (46.10)	69 (48.94)	
	*M (SD)*		
Age	11.72 (2.74)	12.73 (2.77)	>0.05
Weight	46.80 (17.84)	71.06 (22.47)	<0.001
Height	153.24 (17.15)	159.44 (15.34)	<0.01
BMI 2	19.26 (4.31)	27.32 (5.26)	<0.001
BMI z-score 3	0.25 (1.38)	2.34 (0.96)	<0.001

^1^ The number of boys and girls in the two body mass index (BMI) groups did not differ significantly, *χ*^2^ (1, *N* = 282) = 0.23; *p* > 0.05. ^2^ The “OLAF” and “OLA” projects (the Polish growth chart—percentile analysis) were used to calculate children’s and adolescents’ body mass index [14] ^3^ WHO AnthroPlus was used [15].

**Table 2:** Correlations between analyzed variables.

	2	3	4	5	6
1. Negative affect	0.177 **	0.075	0.172 **	0.032	0.009
2. Body dissatisfaction		0.008	0.487 ***	0.559 ***	0.102
3. Uncontrolled eating			−0.125 *	0.154 **	0.168 **
4. Restrictive eating				0.505 ***	−0.073
5. Obesity ^1^					0.087
6. High-energy consumption ^2^					

* *p* < 0.05, ** *p* < 0.01, *** *p* < 0.001; ^1^ dichotomous variable: normal body weight vs. obesity; ^2^ “How often do you eat high-calorie snacks during the day (e.g., sweets, fast food, chips, salt sticks)?”. Participants were asked to respond to the following question by marking their response either: 1—“once a day or less often”, 2—“two times a day”, 3—“three times a day”, 4—“four times a day”, 5—“five times a day”, 6—“six or more times a day”.

**Table 3:** Fit index.

*χ^2^*	*df*	*p*	*χ^2^*/*df*	RMSEA ^1^	*pclose*	SRMR	CFI	AIC ^2^	BIC ^3^
1.60	2	0.449	0.80	0.00	0.655	0.02	0.99	39.60	40.57

^1^ with approximately 90% confidence (0.00; 0.111); ^2^ saturated model: 42.00, independent model: 148.44; ^3^ saturated model: 43.07, independent model: 148.74.

## Abbreviations

AIC	Akaike information criterion
BIC	Bayesian information criterion
BMI	body mass index
CFI	comparative fit index
COD	Circle of Discontent
df	degrees of freedom
DSM-5	Diagnostic and Statistical Manual of Mental Disorders, Fifth Edition
EDY-Q	Eating Disorders in Youth—Questionnaire
GLS	generalized least squares
M	mean
N	sample size
NA	negative affect
p	probability value
PA	positive affect
PANAS-C	Positive and Negative Affect Scale for Children
RMSEA	root mean square error of approximation
SD	standard deviation
SRMR	standardized root mean square residual
TFEQ-R13	Three-Factor Eating Questionnaire
χ2	chi-square statistic
